# The utility of preoperative computed tomography-guided screw marking in thoracic spine surgery

**DOI:** 10.1016/j.bas.2025.104333

**Published:** 2025-07-17

**Authors:** Christopher Marvin Jesse, Aatharshan Kannathasan, Ralph T. Schär, Johannes Goldberg, Andreas Raabe, Jan Gralla, Johannes Kaesmacher, Tomas Dobrocky, Eike Immo Piechowiak

**Affiliations:** a Department of Neurosurgery, Inselspital, Bern University Hospital, and University of Bern, Bern, Switzerland; b Institute of Diagnostic and Interventional Neuroradiology, Inselspital, Bern University Hospital, and University of Bern, Bern, Switzerland

**Keywords:** Spontaneous intracranial hypotension, Spine surgery, Intraoperative fluoroscopy, Wrong-level surgery

## Abstract

**Introduction:**

Wrong-level surgery (WLS) is a preventable yet severe complication in spinal surgery, particularly for pathologies located in the thoracic spine, where localizing the intended level is more challenging compared to the lumbar or cervical spine, which have more distinct landmark structures and fewer vertebral bodies.

**Research question:**

Evaluate the impact of preoperative, computed tomography (CT)-guided screw marking on avoiding WLS and optimizing intraoperative workflows.

**Material and methods:**

We conducted a retrospective case-control study at Bern University Hospital, enrolling all patients treated with thoracic spinal surgery between February 2017 and August 2022. Patients that received preoperative, CT-guided screw marking in the pedicle at the index level were compared to those without preoperative marking. Data included clinical features, radiological parameters, and complications. Primary endpoint: occurrence of WLS. Secondary endpoints: duration of intraoperative fluoroscopy, operating room (OR) occupancy time, and complications.

**Results:**

A total of 117 patients were included: 71 in the screw group and 46 in the control group. The mean age was 54 (±16) years. Significant differences were found in the indication for surgery (p = 0.002). No significant differences were observed in duration of intraoperative fluoroscopy, effective dose, or total OR occupancy time. WLS occurred in only one patient in the control group and none in the screw group. Surgical complications were similar between groups.

**Discussion and conclusion:**

We present a safe technique with a low complication rate for preoperative marking of the index vertebra before thoracic spinal surgery, allowing spine surgeons to eliminate the risk of WLS.

## Highlights

• CT-guided screw marking eliminates wrong-level surgery in thoracic spine cases
• Fiducial screw placement enables precise intraoperative vertebral localization
• Safe technique for screw-marking in thoracic spine surgery with low complication rate

## List of abbreviations

**ASA**: *American Society of Anaesthesiologists*
**CT**: *Computed tomography*
**K-wire**: *Kirschner wire*
**N**: *Number*
**OR**: *Operating room*
**P**: *P-value*
**SD**: *Standard deviation*
**WLS**: *Wrong-level surgery*

## 1 Introduction

Wrong-level surgery (WLS) is a feared yet preventable complication in spine surgery. It ranks among the four most common surgical errors in this field, alongside operating on the wrong patient, performing an incorrect procedure, and operating on the wrong side (Epstein, 2021). WLS occurs when the procedure is not performed on the correct index vertebra where the pathology is located. The reported rate of WLS in the literature ranges between 0.09 and 4.5 per 10,000 interventions (DeVine et al., 2010). As control images are not routinely obtained post-operatively, cases with WLS are probably underreported (Tan et al., 2023). Nevertheless, it is estimated that about 50 % of spine surgeons are involved in at least one WLS during their career (Mody et al., 2008). Thoracic spine surgeries are particularly prone to WLS due to the difficulty in accurately counting vertebral levels to locate the index vertebra. This challenge is further compounded by the frequent need for multiple X-ray frames to count upwards, increasing the risk of miscounting. This is of particular relevance for most intradural pathologies, as they typically do not affect the bony anatomy visible on X-rays, making accurate vertebral level identification even more challenging. WLS is often associated with a higher morbidity and additional surgeries. This not only jeopardizes patient safety but also represents an economic burden for the healthcare system, strains the doctor-patient relationship, and has legal, social and emotional consequences for patients (Chin et al., 2015; Fager, 2006; Goodkin and Laska, 2004). Most important, treating the addressed pathology adequately may not be achieved through WLS.

Nowadays, the correct level is routinely identified by counting bony structures, such as vertebrae and ribs, under intraoperative fluoroscopic control. A cutaneous marking is usually insufficient, since shifting of the soft tissue in relation to the index vertebra, especially during thoracic spine surgery due to thoracic kyphosis, makes such marking unreliable (Upadhyaya et al., 2012). One option is the percutaneous placement of a spinal needle near the spinous process as a marker under fluoroscopic control. However, the accuracy of this method is limited and depends on various factors such as physiological anatomical variations, obesity and image quality (Nowitzke et al., 2008; Tan et al., 2023).

Additionally, a second and final confirmation must be performed using intraoperative fluoroscopy after the skin incision and visualization of the lamina, particularly due to the angulation of the spine, which is especially significant in obese patients with pronounced subcutaneous fat. Thoracic spine surgery can be particularly demanding because of additional obstacles such as superimposition of the shoulders, osteoporosis, and the individual differences in the number of thoracic rib-bearing vertebrae (Upadhyaya et al., 2012). Additionally, index vertebrae of the thoracic spine are regularly distant from both the craniocervical and lumbosacral junctions, making it difficult to capture them together within a single X-ray frame for reference.

Previous studies have presented several methods designed to prevent WLS (Tan et al., 2023). Nevertheless, none of them has become established as a standard method. The preferred method for localization of the intended spine level should be readily accessible, easily reproducible, simply applicable, useable for all forms of spine surgeries, and accurate (Nowitzke et al., 2008). We here describe a method for use in patients with planned thoracic spine procedures who undergo a computed tomography (CT)-guided, percutaneous screw marking in the access pathway prior to surgery, aimed at assisting with accurate intraoperative localization of the intended vertebral level. The aim of this study is to examine the impact of this technique on the reduction of WLS. A further aim was to find out whether an additional screw for simplified intraoperative level localization has an influence on intraoperative parameters such as operating room (OR) occupancy time or duration of fluoroscopy.

## 2 Methods

### 2.1 Study design and patient selection

We conducted a monocentric, retrospective case-control study. Patients who underwent a thoracic spinal neurosurgical procedure between February 2017 and August 2022 at the Department of Neurosurgery of the University Hospital of Bern, Switzerland, were screened for eligibility. We compared a case group consisting of patients with a preoperatively inserted screw marking to a control group comprising patients in whom the localization of the correct level was done under intraoperative fluoroscopic control alone. The screw marking method was introduced and regularly performed as of October 31, 2018 for all patients with an elective thoracic spine surgery as requested by the neurosurgeon. Accordingly, the control group comprises patients treated prior to October 31, 2018, whereas the case group includes patients treated following the introduction of the technique on that date. Only patients who provided a signed informed general consent for the further use of their health-related data were included in this study. Patients without relevant pre-, intra- or postoperative clinical data were excluded from the final analysis. Approval to conduct the study was obtained from the cantonal ethics committee of Bern, Switzerland (approval no. 2023-00923). All procedures were performed in compliance with relevant laws and institutional guidelines.

### 2.2 Data collection

Data were recorded as a part of routine clinical diagnostics and quality assurance and contained information concerning epidemiology and clinical features of patients, radiological parameters (duration and dose of the CT-guided screw marking, duration and dose of intraoperative fluoroscopy), screw intervention (level and location of the screw marking, complications) and surgery (duration, access route, complications). Radiological parameters were obtained from the Sectra Workstation IDS 7 24.2 (Sectra AB, Linköping, Sweden). The remaining data were acquired from the clinical information system. The duration of radiation and the dose applied during the CT guided screw marking were used to assess radiation exposure. The period between the patient entering the operating room and the skin incision (OR occupancy time part 1) includes the time required to identify the correct vertebral level using intraoperative fluoroscopy. This duration will serve as a time metric marker for comparison between procedures performed with and without preoperative screw marking. The time from skin incision to skin closure includes the second confirmatory fluoroscopy and the surgical procedure itself, representing the second phase of operating room occupancy time (OR occupancy time, part 2). The duration of radiation and the dose applied during intraoperative fluoroscopy were incorporated to assess radiation exposure during the surgical procedure.

The primary endpoint of the study was the occurrence of WLS. The correct vertebra corresponded to the closest proximity to the actual location of the spinal pathology, as identified by the neurosurgeon. The secondary endpoints were duration of intraoperative fluoroscopy and OR occupancy time, intraoperative and postoperative complications, as well as complications related to the insertion of the screw.

### 2.3 Technique for CT-guided screw marking

**Fig. 1:**
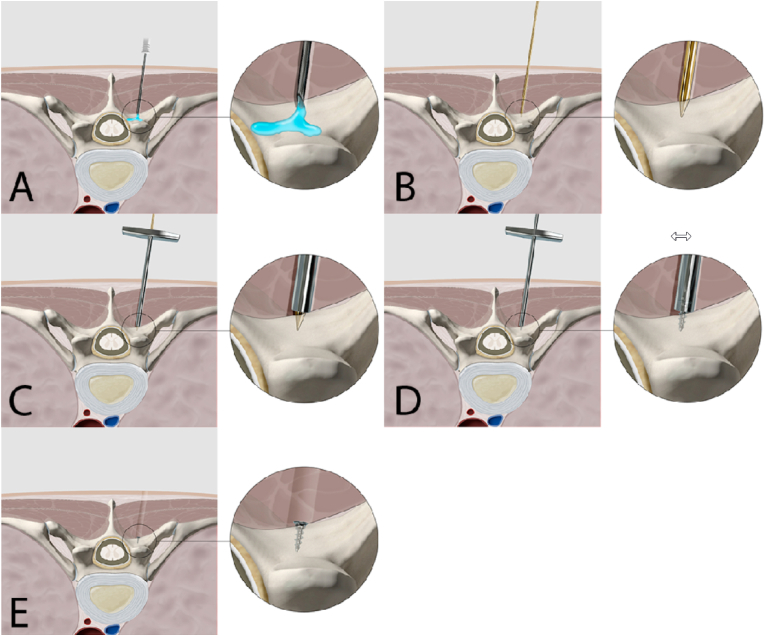
Illustration of the CT-guided, percutaneous screw marking technique. The first step includes local anaesthesia of the skin, soft tissue, and the periosteum (A). Navigation of a K-wire to the index pedicle (B). The trocar is then introduced via the K-wire (C). After removal of the K-wire, the screw is inserted through the trocar into the bone (D). The final result is documented for operation planning (E).

**Fig. 2:**
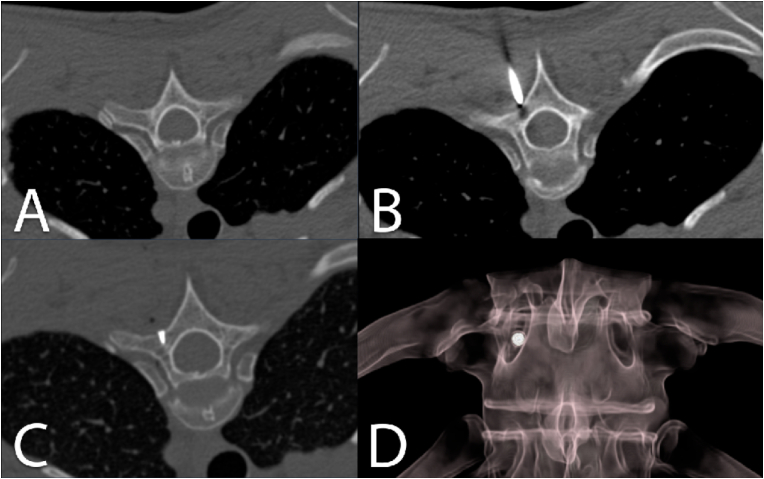
CT image documentation of screw marking in the thoracic spine. CT scan to locate the correct position (A). Positioning of the K-wire at the correct location (B). Documentation of the screw within the surgical trajectory (C). 3D-volume rendered reconstruction with the inserted screw in the index pedicle (D).

In patients undergoing elective surgery at the level of the thoracic spine, a screw marking was performed preoperatively upon request of the primary neurosurgeon. The target for screw marking in most cases was the pedicle entry point of the index vertebra at the most affected side. For this procedure, the patient was positioned prone in the CT-scanner (Somatom Definition Edge or Somatom X.ceed, Siemens Healthcare GmbH, Erlangen, Germany). Under sterile conditions, local anaesthesia of the skin, subcutaneous tissue and the periosteum overlying the index pedicle was performed with 10–20 mL lidocaine 1 %. Under intermittent CT controls a Kirschner wire (K-wire) was advanced to the cortex of the target pedicle. A stainless steel trocar with a diameter of 2.5 mm was then introduced over the K-wire. Once the correct positioning of the trocar had been confirmed a Modus 1.5 SpeedTip 5.5 mm × 0.5 mm screw (Medartis AG, Basel, Switzerland) was inserted through the trocar into the lamina (Fig. 1). The correct positioning of the screw was confirmed by a final CT scan and 3D reconstructions (Fig. 2).

**Fig. 3:**
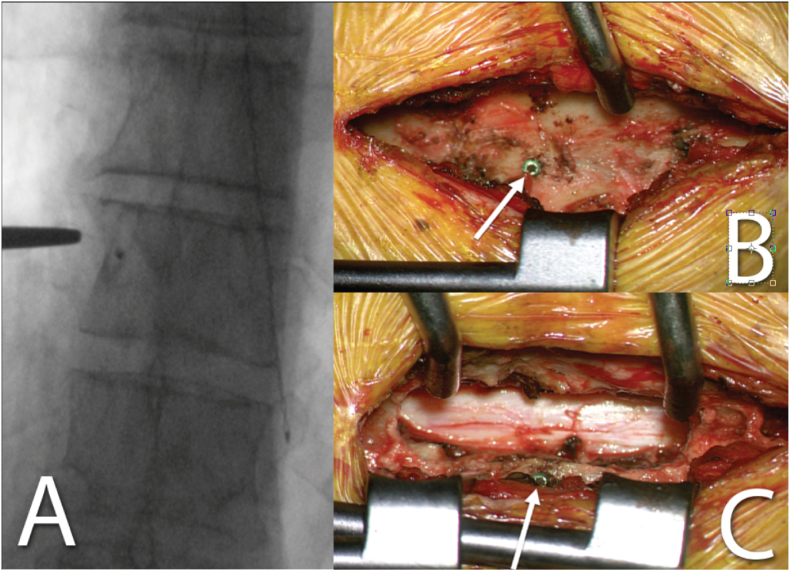
Intraoperative fluoroscopy (A) and intraoperative images showing the inserted screw in relation to bony landmarks before (B) and after left-sided partial hemilaminectomy (C). The arrows point at the screw head.

Before skin incision, a single fluoroscopy image was obtained to verify the index level with the previously inserted screw. After exposure of the lamina, the screw was visually identified, and no further intraoperative imaging was performed (Fig. 3). At the end of surgery, the screw was removed with a screwdriver.

### 2.4 Standard localization technique (control group)

The standard localization technique used in the control group consisted of counting the vertebrae from the craniocervical or lumbosacral junctions in the direction of the index vertebra under fluoroscopic control, using a mobile C-arm system (Arcadis Orbic 3D, Siemens Healthineers AG, Erlangen, Germany or Ziehm Vision RFD, Ziehm Imaging GmbH, Nuremberg, Germany). Fluoroscopic localization was performed before skin incision and after exposure of the bony spinal structures to confirm the correct level.

### 2.5 Statistical analysis

Statistical tests were performed using SPSS Statistics 28.0.1.1 (International Business Machines Corporation, New York, United States of America). A p-value (p) lower than 0.05 was considered significant. The frequencies of the primary endpoint (WLS) between the case and control groups were compared using the chi-square test. To detect a difference in the frequency of WLS between the two groups with a significance level (α) of 0.05, a power of a test (β) 0.8, and a minimum clinical relevance of one patient, we required a sample size of approximately 56 patients in the control group. Since no WLS were assumed in the case group, the sample size in the case group had no influence on the analysis. The secondary endpoints, such as duration of intraoperative fluoroscopy and OR occupancy time, were compared between the case and control groups using Student's t-test. Any complications that occurred during screw marking or during surgery (intra- and postoperatively) were documented.

## 3 Results

### 3.1 Patient characteristics

In total, 117 patients matching the inclusion criteria were enrolled in the final study cohort. Of these, 71 (61 %) received a preoperative CT-guided screw marking. The mean age at surgery in the case and control group were 56 and 51 respectively (p = 0.15). In both groups, 63 % of the patients were female and most patients had an American Society of Anaesthesiologists (ASA) physical status classification of II (34 of 46; 74 %, versus 42 of 71; 59 %). There was a significant difference in indication for surgery (p = 0.002) although tumors and spontaneous intracranial hypotension were the two leading pathologies in both groups. The baseline characteristics are shown in Table 1.

**Table 1:** Baseline patient characteristics.

	Control group (n = 46)	Case group (n = 71)	p-Value
Sex			
Male (%)	17 (37 %)	26 (37 %)	1.0
Female (%)	29 (63 %)	45 (63 %)	
Age at surgery, [years], mean (±SD)	51 (±16)	56 (±16)	0.15
Height, [cm], mean (±SD)	170 (±8.9)	169 (±8.7)	0.59
Weight, [kg], mean (±SD)	71 (±15)	73 (±15)	0.61
ASA classification, n (%)			
I	4 (8.7 %)	5 (7 %)	0.24
II	34 (74 %)	42 (59 %)	
III	8 (17 %)	22 (31 %)	
IV	0 (0 %)	2 (2.8 %)	
Indication for surgery			
Spinal tumor (n)	11	28	0.002
Intrathecal cysts, spinal cord herniation or adhesions (n)	6	9	
Spinal cerebrospinal fluid leaks (n)	28	23	
Vascular pathologies (n)	1	4	
Others (n)	0	7	

n, number; SD, standard deviation; American Society of Anesthesiologists (ASA) classification: I = normal healthy patient, II = mild systemic disease; III = severe systemic disease; IV = severe systemic life-threatening disease.

### 3.2 Radiological parameters and operating room occupancy time

A thorough assessment of the parameters relating to the duration and the effective dose of intraoperative fluoroscopy in the Sectra Workstation IDS 7 24.2 revealed results for only 3 and 2 patients respectively in the control group and for 19 and 18 patients respectively in the case group. There was a slight tendency towards a shorter duration (18 s versus 10 s, p = 0.33) and a lower effective dose (0.11 mSv versus 0.05 mSv, p = 0.32) of intraoperative fluoroscopy in the screw cohort compared to the control group but the differences were not statistically significant (Table 2). Furthermore, no significant difference was observed in the total OR occupancy time between the two groups (199 min versus 216 min, p = 0.26) (Table 3).

**Table 2:** Radiological parameters.

	Control group	Case group	p-Value
Duration of CT-guided screw marking, [min], mean (±SD)	–	25 (±10)	–
Effective dose for CT-guided screw marking, [mSv], mean (±SD)	–	6.8 (±3.7)	–
Duration of intraoperative fluoroscopy, [s], mean (±SD), n (%)	18 (±14), 3 (6.5 %)	10 (±13), 19 (26.8 %)	0.33
Effective dose of intraoperative fluoroscopy, [mSv], mean (±SD), n (%)	0.11 (±0.14), 2 (4.3 %)	0.05 (±0.07), 18 (25.4 %)	0.32

n, number; SD, standard deviation.

**Table 3:** Operating room occupancy time.

	Control group	Case group	p-Value
OR occupancy time part 1, [min], mean (±SD)	36 (±11)	35 (±12)	0.66
OR occupancy time part 2, [min], mean (±SD)	163 (±45)	181 (±87)	0.21
Total OR occupancy time (part 1 + 2), [min], mean (±SD)	199 (±47)	216 (±92)	0.26

OR occupancy time part 1: time between patient arrival in OR and beginning of surgical procedure with skin incision; OR occupancy time part 2: time between skin incision and skin closure.

### 3.3 Complications

One WLS was reported in the control group and none in the case group. Other surgical complications occurred in two patients in the control and five patients in the case group, with secondary epidural bleeding being the leading complication in both groups. No complications related to screw marking were observed in the case group (Table 4). Due to the expected benefits in accurate level localization and the associated increase in safety, all patients accepted the additional intervention and its potential risks.

**Table 4:** Complications.

	Control group	Case group	p-Value
Wrong-level surgery, n (%)	1 (2.2 %)	0 (0 %)	0.39
Total surgical complications, n (%)	2 (4.3 %)	5 (7 %)	0.7
Epidural secondary bleeding	2 (4.3 %)	3 (4.2 %)	
Cerebrospinal fluid fistula	0 (0 %)	1 (1.4 %)	
Other secondary bleeding	0 (0 %)	1 (1.4 %)	
Complications related to screw marking n (%)	–	0 (0 %)	–

n, number.

## 4 Discussion

Our study shows that preoperative CT-guided screw marking is a safe technique to potentially eliminate the risk WLS in the thoracic spine. While intraoperative localization of thoracic pathologies can be challenging, preoperative screw marking is an easy way to facilitate and shorten intraoperative fluoroscopy. No patient in our cohort had any complications related to the screw marking, which underlines the safety of this technique.

Many different techniques that help to prevent WLS in patients undergoing spinal surgical procedures have been described, such as endovascular coiling, fiducials, dye, cement and gadolinium tubes (Tan et al., 2023). However, there is currently no reference standard procedure in clinical practice. Fiducials have often been described in the literature as a viable technique for avoiding WLS (Anaizi et al., 2015; Binning and Schmidt, 2010; Castle-Kirszbaum et al., 2019; Dhahri et al., 2020; Ishak et al., 2020; Madaelil et al., 2017; Maduri et al., 2019; Mongardi et al., 2021; Upadhyaya et al., 2012; Young et al., 2014). Due to their location below the skin and fixation in the bone, disruptive factors prone to lead to WLS, such as body habitus, movement of skin, migration of the marker and space between skin and the vertebra of interest are eliminated. Furthermore, they present a simple practical method with a high success and low complication rate (Upadhyaya et al., 2012). As WLS is more likely to occur in thoracic spine surgeries, these markers have often been tested in the thoracic region (Ahmadi et al., 2014; Anaizi et al., 2015; Binning and Schmidt, 2010; Cornips et al., 2007; Hsu et al., 2008; Ishak et al., 2020; Madaelil et al., 2017; Maduri et al., 2019; Paolini et al., 2005; Thambiraj and Quraishi, 2012; Upadhyaya et al., 2012). Our study sought to explore the utility of a preoperative CT-guided screw marker inserted before thoracic surgery for reducing WLS. A similar study, conducted by Upadhyaya et al., showed that screw marking is effective in the prevention of WLS, but this group used a slightly different technique for inserting the screw (Upadhyaya et al., 2012). Furthermore, our study also looked at whether this procedure has any influence on intraoperative parameters such as OR occupancy time or duration of intraoperative fluoroscopy.

Our findings regarding WLS in the case group are in line with those of Upadhyaya et al. where no WLS was reported among the 26 patients receiving a preoperative screw marking (Upadhyaya et al., 2012). Other studies using different methods of fiducial marking have also reported a correct-level surgery rate of 100 % (Tan et al., 2023). In comparison, one WLS occurred in our control group, accounting for 2.2 % of the patients who underwent surgery. This figure should be considered in the context of the substantial volume of spinal surgeries performed daily in dedicated spine centers. Although wrong-level screw marking cannot be entirely ruled out despite high-resolution cross-sectional imaging, the risk is very low, as demonstrated by the absence of any such cases in our cohort, which is consistent with findings of Upadhyaya et al. (2012). Furthermore, no complications related to screw marking were observed in our study, underlining that the use of CT-guided screw marking is a feasible method to prevent WLS, with a low complication rate, in line with previous studies (Tan et al., 2023).

In our study, the patients in the screw cohort tended to have a slightly shorter duration of intraoperative fluoroscopy. However, this result was not statistically significant (p = 0.33) and the intraoperative data was available only for a minority of patients. In contrast, Upadhyaya et al. reported that the mean fluoroscopy localization time was reduced by 12 min in patients with a preoperative fiducial marking (Upadhyaya et al., 2012). Similarly, Mongardi et al. observed a reduction of the mean duration of fluoroscopy exposure by 12 min in patients with preoperative fiducial marking (Mongardi et al., 2021). However, the radiation exposure associated with CT-guided screw insertion must be considered, and the overall dose is unlikely to be lower in patients undergoing screw marking, except in cases where screw marking could be embedded into the standard preoperative CT protocol.

We expected that the preoperative screw marking would be associated with a shorter OR occupancy time as a result of the straightforward localization of the index vertebra. Unexpectedly, the OR occupancy time tended to be even longer in the case group. However, this result was not statistically significant (p = 0.26). The fact that the indication for surgery differed significantly between the two groups might explain this finding. Certain pathologies necessitate inherently more complicated and time-consuming surgical procedures than others. Tumors and vascular pathologies accounted for a relatively larger proportion of the indications in the case group, whereas the patients in the control group were often treated for CSF leaks. Furthermore, the fact that the OR occupancy time was counted on from patient entering the operating room, duration of time-consuming procedures as positioning and disinfection of the patient was not excluded, which might also have an impact on this finding.

The findings from our cohort do not support the claims of Upadhyaya et al. and Ishak et al. that the placement of a fiducial marker under CT guidance would be cost neutral due to the reduction of OR time. However, that could be due to more complex, time-consuming tumor surgeries were performed in patients in the screw group. Nevertheless, in our opinion, the added costs are outweighed by the advantages of this localization technique, namely elimination of WLS and an optimized intraoperative workflow (Ishak et al., 2020; Upadhyaya et al., 2012).

Our study has several limitations. The generalizability of the results is limited by the study design itself as the retrospective nature of our data make it prone to several biases. Furthermore, the reliability of our results is impacted by the low numbers of patients, despite having a larger patient cohort than in any previous study using fiducial markers. An unequivocal statement on the impact on radiological parameters of intraoperative fluoroscopy was constrained by missing data. An additional limitation of this study is the absence of a formal cost-effectiveness analysis. Nonetheless, we consider the additional expenses to be justified by the potential to prevent wrong-level surgery and its associated clinical and economic consequences. Finally, a proper direct comparison between the case and control group was impossible due to the heterogeneity of the baseline characteristics, especially the difference in indication for surgery.

## 5 Conclusion

We demonstrated a safe and reproducible preoperative screw-marking technique with a low complication rate, specifically designed for intraspinal thoracic spine surgery and well-suited for outpatient settings under local anaesthesia. This approach allows spine surgeons to effectively prevent WLS. Further research with a larger prospective cohort is necessary to validate these findings and assess its impact on operating room efficiency and intraoperative fluoroscopy duration.

## Consent for publication

All authors revised the manuscript and approved the final version to be published.

## Funding

This research did not receive any specific grant from funding agencies in the public, commercial, or not-for-profit sectors.

## Declaration of competing interest

The authors declare that they have no known competing financial interests or personal relationships that could have appeared to influence the work reported in this paper.
